# Medical image segmentation model based on triple gate MultiLayer perceptron

**DOI:** 10.1038/s41598-022-09452-x

**Published:** 2022-04-12

**Authors:** Jingke Yan, Xin Wang, Jingye Cai, Qin Qin, Hao Yang, Qin Wang, Yao Cheng, Tian Gan, Hua Jiang, Jianhua Deng, Bingxu Chen

**Affiliations:** 1 Guilin University of Electronic Technology, School of Marine Engineering, Beihai, 536000 China; 2 University of Electronic Science and Technology of China,School of Information and Software Engineering, Chengdu, 610000 China; 3 China Academy of Engineering Physics, Institute of Applied Electronics, Mianyang, 621900 China; 4 Basic Teaching Department, Guilin University of Electronic Technology, Beihai, 536000 China; 5 Southwest Jiaotong University, State Key Laboratory of Traction Power, Chengdu, 610000 China; 6 Guilin University of Electronic Technology, School of Computer Science and Information Security, Guilin, 541004 China

**Keywords:** Computer science, Biomedical engineering

## Abstract

To alleviate the social contradiction between limited medical resources and increasing medical needs, the medical image-assisted diagnosis based on deep learning has become the research focus in Wise Information Technology of med. Most of the existing medical segmentation models based on Convolution or Transformer have achieved relatively sound effects. However, the Convolution-based model with a limited receptive field cannot establish long-distance dependencies between features as the Network deepens. The Transformer-based model produces large computation overhead and cannot generalize the bias of local features and perceive the position feature of medical images, which are essential in medical image segmentation. To address those issues, we present Triple Gate MultiLayer Perceptron U-Net (TGMLP U-Net), a medical image segmentation model based on MLP, in which we design the Triple Gate MultiLayer Perceptron (TGMLP), composed of three parts. Firstly, considering encoding the position information of features, we propose the Triple MLP module based on MultiLayer Perceptron in this model. It uses linear projection to encode features from the high, wide, and channel dimensions, enabling the model to capture the long-distance dependence of features along the spatial dimension and the precise position information of features in three dimensions with less computational overhead. Then, we design the Local Priors and Global Perceptron module. The Global Perceptron divides the feature map into different partitions and conducts correlation modelling for each partition to establish the global dependency between partitions. The Local Priors uses multi-scale Convolution with high local feature extraction ability to explore further the relationship of context feature information within the structure. At last, we suggest a Gate-controlled Mechanism to effectively solves the problem that the dependence of position embeddings between Patches and within Patches in medical images cannot be well learned due to the relatively small number of samples in medical images segmentation data. Experimental results indicate that the proposed model outperforms other state-of-the-art models in most evaluation indicators, demonstrating its excellent performance in segmenting medical images.

## Introduction

Image segmentation is critical in the medical field and plays a vital role in computer-aided intelligent diagnosis. Medical image segmentation research has been applied to many clinical tasks, such as COVID-19 screening, polyp segmentation, colonoscopy, etc. Early medical image segmentation technologies are mainly threshold or boundary-based segmentation methods^1^. With the development of deep learning, Convolutional Neural Networks (CNNs) have been applied to image denoising^2^ and medical segmentation^3^, etc. In the medical image segmentation field, many novel models have been raised, such as U-Net^4^ proposed by Ronneberger et al., and Res-UNet^5^ proposed by Xiao et al., which extracts feature information by down-sampling and obtains feature information of different scales by up-sampling and jump connection. They are designed specifically for medical segmentation and have achieved good results. However, CNN has made great contributions to medical image segmentation, but it is hard to make any further breakthroughs. Although Convolution can focus on the sub-partitions of the image, it only focuses on local features and loses the global context features, and cannot establish long-term dependence between features because of its inherent bias when extracting features. Convolution’s continuous stacking and down-sampling operation can increase the model’s receptive field and enable Convolution to extract the interactive information between local features, but this method will make the model more complex and prone to overfit.

At present, some studies have modelled the long-distance dependence between features, such as the Attentional mechanism^6^ and Transformer^7^. However, the Attention mechanism is not available in medical image segmentation because it is highly dependent on external information and cannot capture the internal correlation of data or features. Therefore, the Attention mechanism still needs to be further improved. Recently, the Transformer architecture has been widely discussed in medical segmentation tasks. TransUNet^8^ proposed by Chen et al., uses the Transformer to encode the feature map extracted by CNN and uses the extracted global context information to model remote dependency. ViT^9^-based TransFuse^10^ proposed by Zhang et al., together with Transformer and CNN, can improve the efficiency of global context modelling without losing the ability to locate low-level details. Although the above models based on Transformer show great potential in medical image segmentation, they have the following shortcomings:

- Transformer enhances the model’s ability to extract global features through multi-head Attention but does not increase the Local Priors. The lack of Local Priors to summarize feature biases between data requires many training data to make the model converge.
- Part of medical images have fixed position priors, and the multi-head Attention in Transformer does not share parameters of all positions, limiting the use of position information. For example, the human liver, which is in the lower part of the heart and the upper right part of the stomach, needs to be segmented for detection. Convolution in Transformer cannot fully use the parameter to share the heart, stomach, and liver information for position perception.

**Figure 1:**
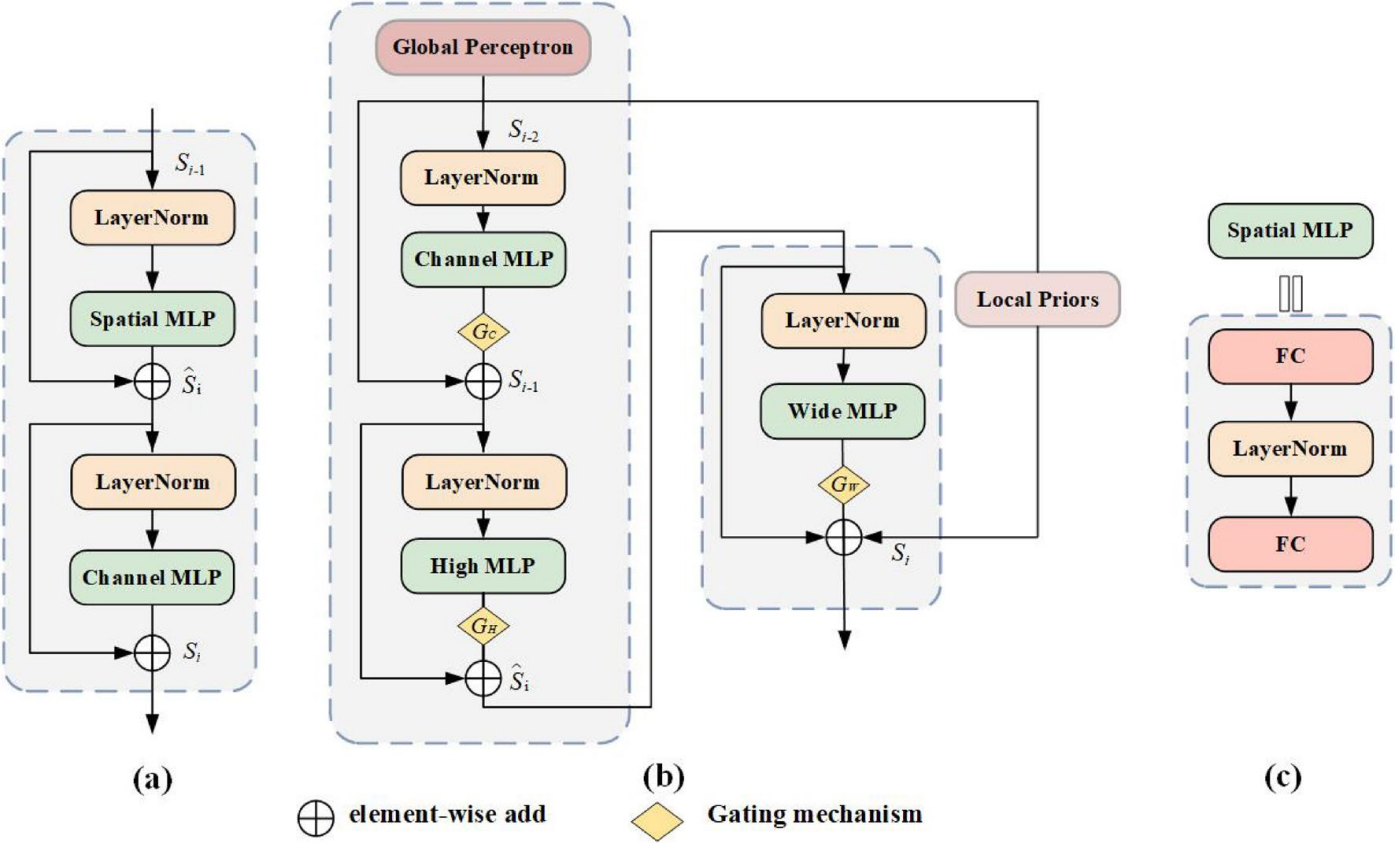
(**a**) is the MLP-Mixer architecture (formulas 1 and 2). (**b**) is the TGMLP architecture (formulas 3, 4 and 5). (**c**) is the spatial MLP architecture wherein Spatial MLP contains two fully connected Layers. (High MLP , Channel MLP and Wide MLP contain only one full connection, respectively).

Recently, Tolstikhin et al. proposed the MLP-Mixer model^11^ based on MLP, which uses full connections to flatten feature maps along the channel axis and spatial axis and encodes them so that the MLP-Mixer can model global context information relationships, as shown in Fig. 1a. Although the MLP-Mixer is more efficient in modelling global context information than Convolutional Neural Networks and Transformer, MLP-Mixer has the following weaknesses:

- The MLP-Mixer linearly projects and encodes spatial information along the spatial dimension, as shown in Fig. 1c, which destroys the feature structure in the original space dimension, resulting in the loss of position information carried by three-dimensional features and the computation overhead increasing quadratically.
- The MLP-mixer uses a full connection to replace Convolution, leading to the loss of spatial information of small-scale objects in feature maps and the lack of local prior features.
- The performance of the MLP-Mixer largely depends on the scale of training DataSets. Without large-scale training DataSets, its performance is still inferior to CNN and Transformer.

To address the above problems, we propose a new model in medical image segmentation in this paper: TGMLP U-Net. In this model, we design the TGMLP, as shown in Fig. 1b, composed of three parts. Firstly, a TM module is advanced, consisting of three independent branches, and each branch encodes along a specific dimension (high, wide, or channel dimension). It can preserve the feature structure in the original space dimension of the input feature map, enable the model to generate long-distance dependencies, generate specific position information in three dimensions, and reduce the computation overhead from quadratically increasing caused by encoding along spatial dimension to linearly increasing. Besides, LGP is proposed. The Global Perceptron divides the feature map into different partitions. It transmits them into multiple Full-Connection Layers(FC) to share the parameters among different partitions, reducing the loss of small-scale feature information in segmenting medical images and modelling global context more effectively. Meanwhile, the Local Priors constructs the CNN and Batch Normalization (BN) parallel to FC. CNN and BN are used to extract local features, avoiding the loss of local feature correlation caused by feature splitting. Finally, as the trained images with corresponding labels in medical DataSets is relatively rare and the cost of making labels is also high, we design a Gate-controlled Mechanism in this paper. The Gate is a learnable parameter, making the proposed mechanism applicable to DataSet of any size. According to the size of the DataSet, the Gate-controlled Mechanism will know whether the number of images is sufficient to learn the correct position for embedding. Depending on whether the information learned by position embedding is useful, the Gate parameters will either converge to 0 or a higher value. To sum up, the contributions of this paper are as follows:

- We design a TM module, a new structure to encode spatial feature information. It can encode spatial feature information along the high, wide and channel dimensions, generate long-distance dependence, and perceive position sensitively while preserving the feature structure in the original space dimension of the input feature map with little computational overhead.
- We introduce the LGP module to complementarily extract local and global features, making the model perceive features in small-scaled and complex objects better and extract features better.
- We present the Gate-controlled Mechanism applicable to DataSets of different sizes, making it easier to learn the position bias of feature maps in medical image DataSets of different sizes.
- Experimental results illustrate that the proposed model excels other state-of-the-art models in most evaluation indicators, demonstrating its outstanding performance in segmenting medical images.

## Related work

In this section, first of all, we summarize the typical Convolutional Neural Network-based methods in medical image segmentation. Then, we review the application of Transformer, especially in medical segmentation. Finally, we list the existing MultiLayer Perceptron (MLP) methods and compare them with the proposed model.

### Convolutional neural network-based model

Convolutional Neural Networks have been widely used in medical image segmentation and show excellent performance. UNet++^12^ proposed by Zhou et al. uses many dense skip structures to extract multi-scale features and reduce semantic gaps. Attention U-Net^13^ proposed by Oktay et al., utilizes the Attention mechanism to intensively extract significant features useful for specific tasks (such as relevant tissues or organs) from medical images and suppress the irrelevant input. Res-UNet adds a weighted Attention mechanism to the U-Net for retinal vascular segmentation, enabling the model to learn more features to distinguish vascular and non-vascular pixels and better maintain the retinal vascular tree structure. In order to capture more semantic information, the DoubleU-Net^14^ proposed by Jha et al. uses Atrous spatial pyramid pooling^15^ (ASPP) to perform parallel sampling with the empty Convolution of different sampling rates for the given segmented image. This method can capture the context information from multiple scales and improve the segmentation performance. UNet 3+^16^ proposed by Huang et al. adopts full-scale jump connection and deep supervision to learn useful information from multi-scale information. Those Convolutional Neural Network-based models mentioned above are featured by inherent inductive bias, such as translation invariance and local connectivity, which is helpful for image feature extraction. However, they mainly focus on the correlation between two-dimensional local data. The attention area will be wider only with the Network Layer deepening, making the model unable to fully use the position information and extract the three-dimensional global spatial information from the feature map.

### Transformer-based model

The Transformer is widely used in the NLP field. Only in recent years, Transformer has been applied to computer vision tasks. The Vision Transformer (ViT)^9^ proposed by Dosovitskiy et al. applies Transformer directly into image processing and achieves brilliant results on the imagenet-21K DataSet. In medical image segmentation, TransUNet uses Transformer to label feature images from CNN as image blocks and encode them to extract the input sequence of the global context. Feature maps encoded by Transformer will be up-sampled, and the up-sampled feature maps will be fused with those extracted by high-resolution CNN to achieve accurate positioning. TransFuse fuses CNN and Transformer to obtain the feature’s global dependence and low-level spatial details in medical images. To effectively train the model on the medical image DataSet, Valanaras et al. put forward Medical Transformer (MedT)^17^, which utilizes Axial Attention^18^ and a Gate-controlled Mechanism to control weight distribution and achieves better performance. Combining the characteristics of Convolution and Transformer, Li et al. raise a Dual-Coding Network based on X-Net^19^, which can extract global and local features by using Convolution and Transformer to achieve a good segmentation effect. Different from the above models, the TGMLP structure proposed by us replaces the Transformer with a MLP structure, which reduces the computational complexity of the Transformer from the square calculation to the linear calculation of the input image size and eliminates the influence of position-perception limitations of multi-head Attention in Transformer. The TGMLP structure can also alternately implement information exchange between Patches and within Patches in the feature map to improve segmentation efficiency.

### MultiLayer perceptron-based model

Compared with single-layer perceptron, multiLayer perceptron (MLP) adds a hidden layer to solve the problem of nonlinear separability. Its neurons in each layer are fully connected, endowing MLP with good parallel processing ability, fault tolerance ability, adaptive learning ability and memory ability. In addition, compared with Convolution, MLP is mainly characterized by full connectivity, which is different from the local connectivity of the Convolution layer. With those advantages, MLP has gradually become the research focus. For example, MLP-Mixer can encode spatial information and performs better, but it requires a large-scale DataSet for training to achieve good results. The ResMLP^20^ proposed by Touvron et al. uses flattened image blocks as input, adopts the linear layer to project the input, and then uses the residual operations to update the projected features. Finally, the obtained feature blocks are classified after average-pooling. The training of ResMLP is more stable than that of Transformer and CNN. Liu et al. ’s newly proposed Attention-Free Network gMLP^21^ controls the amount of information in feature maps only with Gating MLP, making the performance of MLP comparable to Transformer in language and visual tasks. However, these models input three-dimensional spatial features of images two-dimensionally for linear projection, which cause the loss of some position information and feature information of small-scale objects and the lack of local prior features. Different from the above research, the TGMLP presented by us can encode three-dimensional spatial features based on MLP, together with a LGP module and a Gate-controlled Mechanism, making the model more sensitive to the position information in the feature map and capable of modelling long-term dependencies and encoding local prior features more accurately. The model we explored is suitable for medical image segmentation, as the tissue structures of the body in medical images are often very different and complex, and the DataSet for segmentation is relatively small.

## Methods

This section introduces the proposed Triple Gate MultiLayer Perceptron U-Net (TGMLP U-Net) model for medical image segmentation exhaustively. Specifically, we briefly introduce the basic architecture of the model; then, we describe its main components in detail: Triple MLP (TM) structure, Local and Global Perceptron, and Gate-controlled Mechanism.

### Triple gate multilayer perceptron U-net

**Figure 2:**
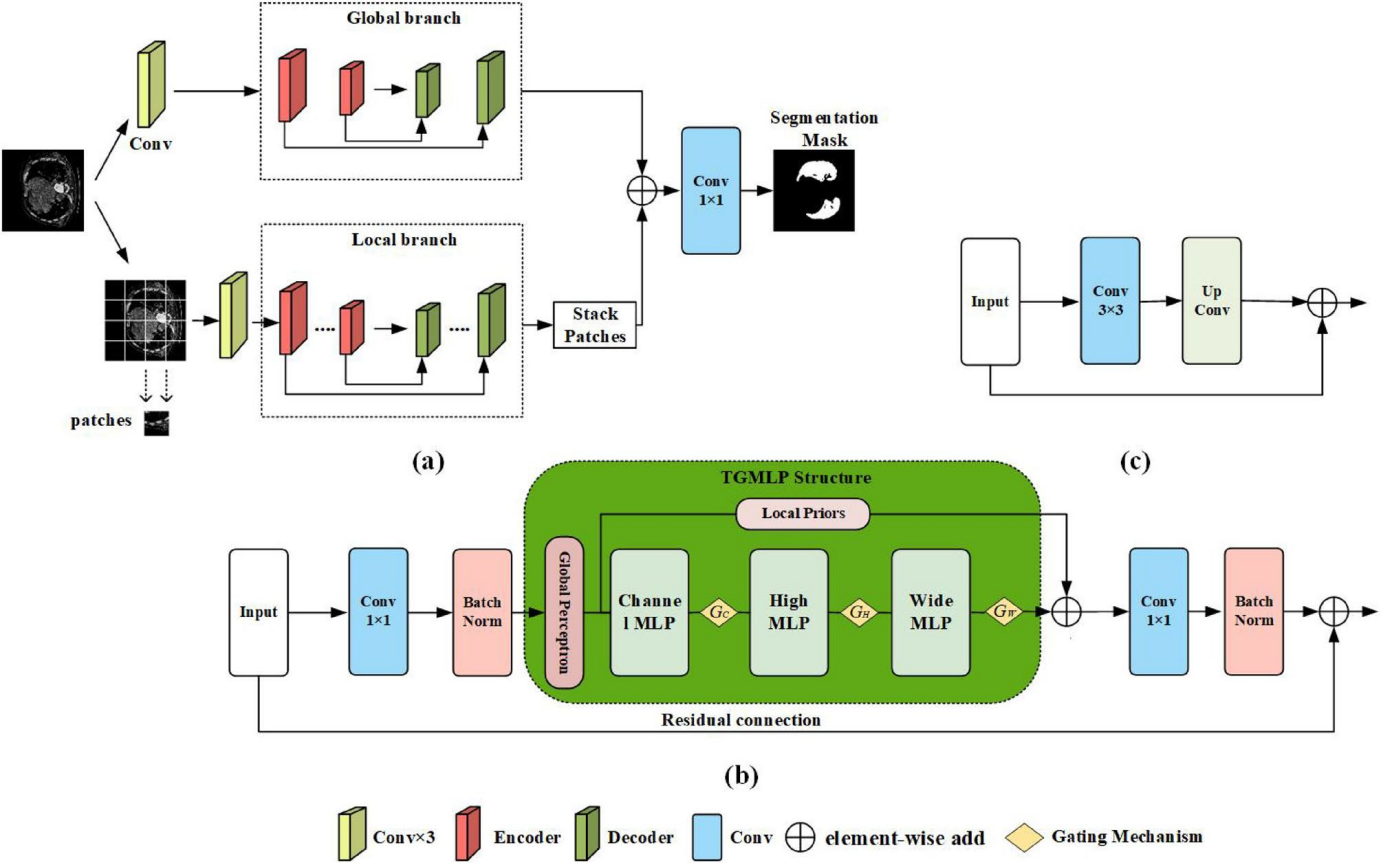
(**a**) shows the main architecture of the TGMLP U-Net with local-global training strategy. (**b**) Indicates that Encoder in TGMLP U-Net uses TGMLP and Convolution. (**c**) indicates that Decoder in TGMLP U-Net uses $3\times 3$ Convolution layer, Deconvolution and jump connection (the Layer Norm and residual connection are omitted in Fig. 2b).

TGMLP U-Net uses TGMLP as the basis and a Local-global training strategy. In the medical image segmentation task, the segmentation mask is larger than the Patch size, limiting the model to learn the feature information and dependence of pixels between Patches. Our model adopts a two-branch structure based on the Medical Transformer^17^ suggested by Valanarasu et al. to make the model better understand the medical image. The two-branch structure includes local branch structure and global branch structure. The global branch is mainly used to learn the long-distance feature relationship, and the local branch can make up for the lost local detail features between Patch pixels. When TGMLP U-Net predicts, it firstly stacks the features of all Patch blocks output by local branches, then uses the add function to add the feature maps extracted from global branches and local branches, and finally uses $1\times 1$ Convolution layer to classify feature maps at the pixel level. Before entering the two-branch structure, the medical image will go through three Convolutional Layers for initial feature extraction, and each Convolutional layer has a normalization and Relu activation function. The Encoders of the two branches are composed of a Convolution layer and TGMLP structure. The Encoder makes the image features gradually reduced and abstracted while the Decoder gradually restores the output of the Encoder to the original size, classifies pixel by pixel, and obtains the segmented image. In the Encoder of TGMLP U-Net, TGMLP encodes feature maps along the high axis, wide axis and channel axis, respectively. Then the Global Perceptron and the Local Priors are incorporated into the TGMLP structure, which not only enables the model to conduct modelling for the global context information of the feature map and establish the external dependencies between the global features but also enables the model better to extract the local information of the feature map. Finally, TGMLP adds a Gate-controlled Mechanism to control the output information and retain the feature information in the feature map to the maximum extent. The encoded features output by TGMLP will be connected with a $1\times 1$ Convolution layer, and the features after Convolution will be connected with residual mapping. Then add function is used to add the features after Convolution and the features input into TGMLP to obtain the final encoded feature map. Figure 2b indicates the architecture of TGMLP and Convolution in the Encoder. The Decoder comprises a $3\times 3$ Convolution layer, Deconvolution and jump connection. The Convolution in the Decoder is to reduce the number of channels in the feature map, while the Deconvolution is to increase the size of the feature map in turn. The Deconvolution results in the decoding part and the output of the encoding part are correspondingly connected and combined by jump connection, recovering feature information gradually. Figure 2c shows an overview of the Decoder. There are two Encoders and two Decoders in the global branch of the TGMLP U-Net. There are five Encoders and five Decoders in the local branch. The overall architecture of GMLP U-Net is shown in Fig. 2a.

### Triple MultiLayer perceptron

As Fig. 1a shows, the recent MLP-Mixer is composed of two different MLP blocks: Channel MLP and Spatial MLP, responsible for encoding channel information and spatial information, respectively. Each MLP of MLP-Mixer is preceded by a LayerNorm (LN) layer and a residual connection. The output ${S_i}$ of MLP-Mixer at the *i*th layer is expressed as:

1 $$\begin{aligned}&{{\widehat{S}}_{\mathrm{{i}}}} = ChannelMLP\left( {LN\left( {{S_{i - 1}}} \right) } \right) + {S_{i - 1}}, \end{aligned}$$

2 $$\begin{aligned}&{S_i} = SpatialMLP\left( {LN\left( {{{{\widehat{S}}}_{\mathrm{{i}}}}} \right) } \right) + {{\widehat{S}}_{\mathrm{{i}}}}, \end{aligned}$$

According to formulas (1) and (2), MLP-Mixer calculates the global affinity and models the global context information in a long-distance dependency way. Different from Convolution, MLP-Mixer can capture non-local information from the entire feature mapping. The spatial MLP in MLP-Mixer needs to calculate the spatial relationship between Patches, whose computational complexity is $\mathrm{O}({H^{_2}}{W^{_2}})$ ,making MLP-Mixer unapplicable to segment medical images with high density and high resolution. In addition, different from Convolution, when calculating local feature information, MLP-Mixer uses spatial MLP to encode two-dimensional spatial feature information, which is not conducive to obtaining the position information. However, position information is crucial in medical image segmentation and is often used to help extract the structure of the segmented object. For efficient and accurate modelling, the TGMLP proposed by us is projected along the feature map’s high axis, wide axis and channel axis, as shown in Fig. 1b. With better computation efficiency, this method reduces the computation complexity of TGMLP to $\mathrm{O}(HWC)$ . It also makes the model more sensitive to the position information, which can capture the remote interaction with accurate position information, and can encode the spatial structure of medical images. Therefore, for a given input feature mapping $x \in {R^{{C_{in}} \times H \times W}}$ with height *H*, width *W*, and channel ${C_{in}}$, the TGMLP output ${S_i}$ of the ${i^{th}}$ layer with high axis, wide axis and channel axis is expressed as:

3 $$\begin{aligned}&{S_{\mathrm{{i - 1}}}} = ChannelMLP\left( {LN\left( {{S_{i - 2}}} \right) } \right) + {S_{i - 2}}, \end{aligned}$$

4 $$\begin{aligned}&{{\widehat{S}}_{\mathrm{{i}}}} = HighMLP\left( {LN\left( {{S_{i - 1}}} \right) } \right) + {S_{i - 1}}, \end{aligned}$$

5 $$\begin{aligned}&{S_i} = WideMLP\left( {LN\left( {{{{\widehat{S}}}_{\mathrm{{i}}}}} \right) } \right) + {{\widehat{S}}_{\mathrm{{i}}}}, \end{aligned}$$

### Local priors and global perception

**Figure 3:**
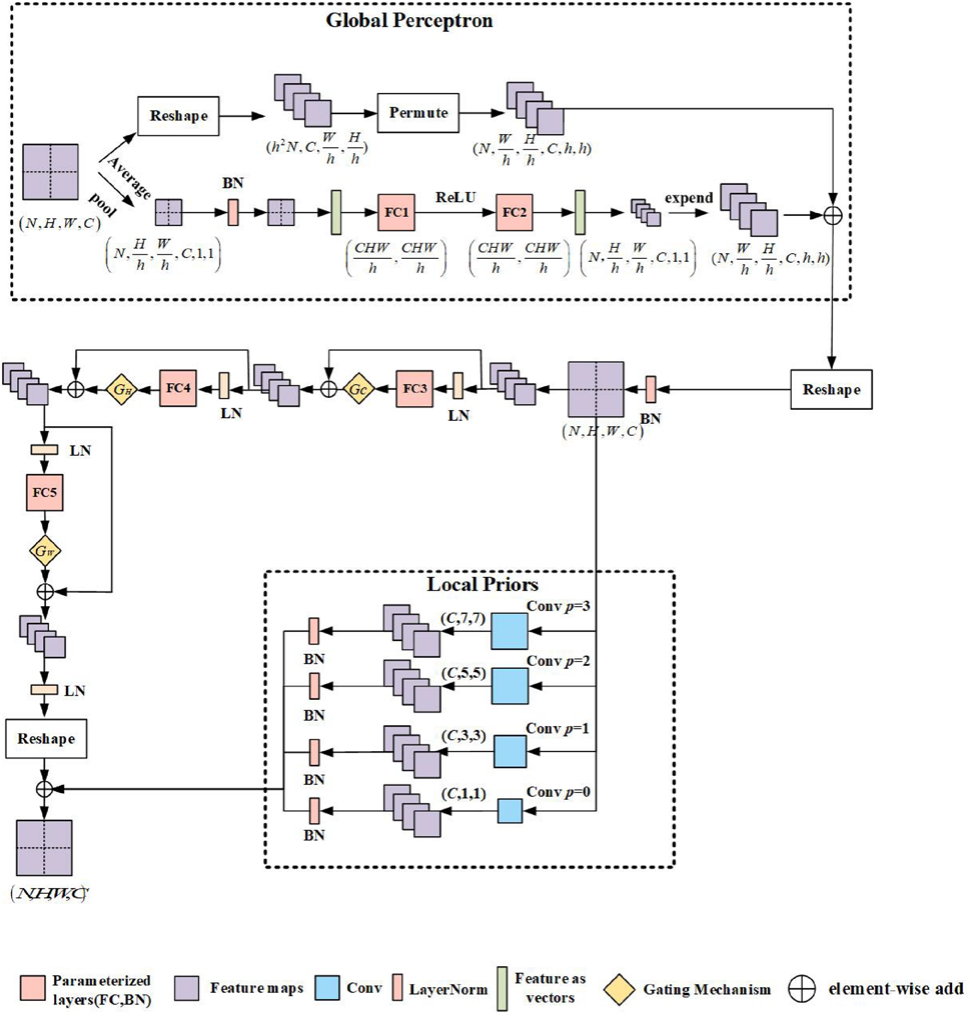
The TGMLP structure is described exhaustively in the figure where *N*, *C*, *H*, and *W* represent the batch size, channel number, high, and wide of the feature map, respectively, p represents the pixel to be filled, and h represents the size of the segmented area of the feature map. Global Perceptron adds a dependency weight matrix to each partition. Local Priors capture prior local features of feature maps through multiple parallel Convolutions.

TMLP uses full connections as a feature extractor to extract fine-grained and coarse-grained features from medical images. However, when segmenting medical images, full connection extracts feature from a partition, prone to ignore small-scale objects such as cells, and cause the loss of local details within the structure. To solve these problems, based on the RepMLP model^22^ proposed by Ding et al., we incorporate Local Priors and Global Perceptron into TMLP. The Global Perceptron divides the feature map into different partitions and uses the shared parameters of different partitions to more effectively model global context information and establish external dependencies between global features. The Local Priors mainly uses Convolution to obtain local features better to preserve the internal dependence between features. The TMLP structure is shown in Fig. 3.

The Global Perceptron divides the feature map into different partitions and makes them share parameters. The way we use to divide partitions is as follows: First, the feature map with the input size of $x \in {R^{N \times C \times H \times W}}$ is divided into *h* partitions; the size of the feature map is reset to $({h^2}N,C,\frac{W}{h},\frac{H}{h})$ ; the axes are sorted again, and the size of the feature map becomes $(N,\frac{W}{h},\frac{H}{h},C,h,h)$ , as shown in Formula (6).

6 $$\begin{aligned} {x^{out}} = P\mathrm{{er}}mute(RS\left( {{x^{in}},({h^2}N,C,\frac{W}{h},\frac{H}{h})} \right) \mathrm{{ )}}, \end{aligned}$$

Where *RS* stands for a function that changes the shape specification of the tensor without changing the order of data in memory; Permute means to reorder the axes of the feature map. Then the global average pooling operation is used to get a matrix of size $(N,\frac{W}{h},\frac{H}{h},C,1,1)$ and input the matrix into BN and a two-layer MLP to get a weight matrix of size$(N,\frac{W}{h} \times \frac{H}{h} \times C)$ , as shown in Formula (7).

7 $$\begin{aligned} {V^{out}} = MLP(BN(GAP\left( {{x^{\mathrm{{out}}}}} \right) )), \end{aligned}$$

Where *GAP* denotes global average pooling, *W* represents Convolution kernel, and *MLP* represents $RS\left( {{x^{out}},(N,C \times \frac{W}{h} \times \frac{H}{h})} \right) \times W(1 \times \frac{W}{h} \times \frac{H}{h},C \times \frac{W}{h} \times \frac{H}{h})$ .To achieve correlation between different partitions of the same channel, we first reset the weight matrix to $(N,\frac{W}{h},\frac{H}{h},C,1,1)$ ; then use the Expend function in Pytorch to change the weight of the matrix to $(N,\frac{W}{h},\frac{H}{h},C,h,h)$ ; finally we use the Add function to add the weighted matrix to each partition to get the feature map ${M^{out}}$ with the size of $(N,\frac{W}{h},\frac{H}{h},C,h,h)$ , as shown in Formula (8). The Global Perceptron realizes the correlation between each pixel and different partitions, offsetting the loss of small-scale objects in feature extraction.

8 $$\begin{aligned} {M^{out}} = \exp end(RS\left( {{V^{out}},(N,\frac{W}{h},\frac{H}{h},C,1,1)} \right) \mathrm{{) + }}{x^{in}}, \end{aligned}$$

The Local Priors module first changes the tensor’s shape specification output by Global Perceptron into $\left( {N,H,W,C} \right)$ and constructs four parallel Convolutional Layers; a BN layer follows each Convolutional layer. Then, the tensor with changed shape specification is input into four parallel Convolutional Layers. The four parallel Convolutional Layers solve the problem of local structural feature loss in feature extraction. The Convolution kernels’ sizes of the four Convolutional Layers are 1,3,5,7, respectively. The padding of the Convolution is used to ensure resolution ($p=0,1,2,3$). Finally, outputs of all Convolution branches are added with those of TMLP to obtain the final output. The calculation method of Local Priors is shown in Formulas (9) and (10).

9 $$\begin{aligned} {U^{out}}= & {} RS\left( {{M^{out}},(N,W,H,C)} \right) , \end{aligned}$$

10 $$\begin{aligned} {x^{out}}= & {} \sum \limits _{i = 1}^4 {Conv\left( {{U^{out}},({F_i},{P_i})} \right) } \mathrm{{ + }}{S_i}, \end{aligned}$$

Where *F* represents the Convolution kernel of the four Convolution Layers, they are 1,3,5,7 respectively; *P* represents the number of pixels that the Convolution layer uses to fill, they are 0,1,2,3 respectively. ${S_i}$ is the value of formula (5).

### Gate-controlled mechanism

We have discussed the benefits of applying TMLP, LGP in medical image segmentation. They enable TGMLP to calculate global context feature information with good computation efficiency and encode remote interactions within the input feature mapping. However, TGMLP is more likely to learn position bias when evaluated on an extensive medical DataSet. Position bias is challenging to learn in experiments conducted on small-scale medical image DataSets, so encoding remote interactive position information is inaccurate. When the learnt position bias is not accurate enough, TGMLP with TMLP cannot give full play to the performance of TMLP. Therefore, we explore a TMLP with the Gate-controlled Mechanism to control the influence of position bias on local position perception. After modifying TMLP, we apply the Gate-controlled Mechanism the high axis of the TMLP, as expressed by Formula (11). The Gate-controlled Mechanism applied to the wide axis and channel axis of TMLP is the same as formula (11).

11 $$\begin{aligned} {{\widehat{S}}_{\mathrm{{i}}}} = {G_H}High MLP\left( {\mathrm{{LN}}\left( {{S_{i - 1}}} \right) } \right) + {S_{i - 1}}, \end{aligned}$$

Among them, the Gate-controlled Mechanism is added to the formulas (3), (4), and (5) of TMLP, they are ${G_C},{G_H},{G_W} \in R$ , respectively. They are learnable parameters and jointly construct the Gate-controlled Mechanism. Usually, if the model accurately learns the position-coded information, the Gate-controlled Mechanism will assign high position weights to the axis of the TMLP.

## Experiments and analysis

In this section, we first introduce the basic information of three medical image segmentation DataSets, evaluation indicators, implementation details, etc. Then, we compare our model with other state-of-the-art models on three public DataSets and visualize part of the results to evaluate the learning and generalization abilities of TGMLP U-Net. Finally, we conduct an ablation experiment on the COVID-19 DataSet^23^^24^.

### Experimental settings

#### DataSets

COVID-19 DataSet, Polyp Segmentation DataSet^25^, and MoNuSeg DataSet^26^ are used for the experiment in this paper. The COVID-19 DataSet contains 100 axial CT images from more than 40 COVID-19 patients. The ground-glass lung nodules, pulmonary consolidation and pleural effusion in the images of this DataSet are the main evidence of novel coronavirus infection. The Polyp Segmentation dataset is a public DataSet published by the International Conference on Medical Image Computing and Computer-Assisted Intervention (ICMA) in 2015, containing 612 colonoscopy images and corresponding semantic labels for colon polyps. The MoNuSeg dataset is a small cell nucleus dataset with pixel-level annotations, containing 30 image training sets and 14 test sets. It includes four types of nuclei, including the inflammatory nucleus and fibroblast nucleus.

#### Evaluation indicators

To compare our model with the state-of-the-art models, we use standard evaluation indicators, including Mean Dice Coefficient (mDice), Hausdorff Distance (HD), Mean Intersection over Union (mIoU), Precision, Recall, Sensitivity (SEN), Specificity (SPC), among which the values of mDice, HD, mIoU, precision, recall, SEN range between 0 and 1. The lower the HD value, the higher the segmentation accuracy. The larger the other values, the better the segmentation performance. In addition, we also test the model’s Parameters and FLOPs. the smaller the Parameters and FLOPs, the better the model’s performance.

#### Loss Function

In the model’s training, we make comparative experiments on the Loss Functions based on Dice Coefficient, Cross-Entropy and Focal Loss, as shown in Table 5. After calculating the Loss Function, we notice that the Cross-Entropy Loss Function can achieve the optimal results. Therefore, it is adopted in this paper, as shown in Formula (12).

12 $$\begin{aligned} \begin{array}{l} {{{\mathcal{L}}}_{CE(p,{\hat{p}})}} =-\frac{1}{{wh}}\sum \limits _{x = 0}^{w - 1} {\sum \limits _{y = 0}^{h - 1} {(p(} } x,y)\log ({\hat{p}}(x,y))) \mathrm{{ }} + (1 - p(x,y))\log (1 - {\hat{p}}(x,y)) \end{array}, \end{aligned}$$

Where *w* and *h* are the image’s sizes,*p*(*x*, *y*) is the label corresponding to the pixel of the image, and ${\hat{p}}(x,y)$ represents the model’s output prediction probability . It should be noted that the calculation method of Focal Loss in the comparative experiments is shown in Formula (13).

13 $$\begin{aligned} \begin{array}{l} {{{\mathcal {L}}}_{FC(p,{\hat{p}})}} = - \frac{1}{{wh}}\sum \limits _{x = 0}^{w - 1} {\sum \limits _{y = 0}^{h - 1} {\alpha {{(1 - p(x,y))}^\gamma }\log {\hat{p}}(x,y) + (1 - \alpha )p{{(x,y)}^\gamma }{{\log }_a}(1 - {\hat{p}}(x,y))} } \end{array}, \end{aligned}$$

Where the larger the value of $\alpha$ is, the larger the Loss Value contributed by the object to be segmented will be. $\gamma$ represents the regulating factor to reduce the weight of the background, making the model focus on segmenting the objects in medical images. In our experiments, we set $\alpha$ as 0.25 and $\gamma$ as 2, respectively.

#### Experimental parameters

The experimental configuration is listed as follows: video card: NVIDIA GeForce RTX 3070Ti, the processor: Intel i7-8700 CPU, the memory: 16G, the software platform: Windows 10 Pytorch 1.8 and Python 3.6. We finally select a group of experimental parameters with the best experimental results by constantly adjusting the experimental parameters. The input image size is fixed at $128\times 128$, and the iteration times of training are 400 generations. The optimizer is set to Adam with a batch size of 10. The initial learning rate of the Adam algorithm is set to 0.001, and the minimum learning rate is set to 0.00001. The attenuation strategy of the learning rate is cosine annealing, and warmup epochs is set to 100. To speed up the model’s convergence, we carry out batch normalization for each Convolution layer with epsilon as 0.00000 1 and momentum as 0.1. To ensure the fairness of the comparison, we use the same training settings for all the models involved in this experiment.

### Experimental results

#### COVID-19 DataSet

**Figure 4:**
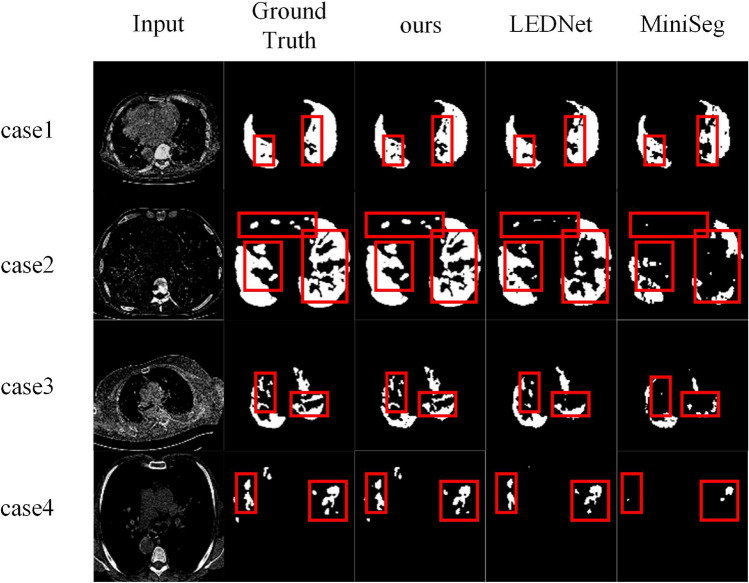
shows the qualitative results of TGMLP U-Net in COVID-19 tasks versus results of other models. TGMLP U-Net with better learning and generalization abilities performs better.

As shown in Table 1, most indicators of the TGMLP U-Net are superior to those of other advanced models on the COVID-19 DataSet. To be specific, the values of mIoU, SEN, SPC, DSC, HD of TGMLP U-Net reach 86.45%, 91.20%, 98.31%, 89.06% and 50.94%, respectively. Compared with MiniSeg^27^, the values of mIoU, SEN, SPC, DSC of TGMLP U-Net increase by 4.3%, 9.25%, 0.6%, 13.15%, respectively. HD decreases by 23.48%. As shown in Fig. 4, the segmentation effect of TGMLP U-Net excels other models. Its results are close to the true values, and there are fewer segmentation errors in tissue partitions. The results of LEDNet^28^ are not satisfactory as there are many unsegmented tissues. MiniSeg has better results but is still not satisfactory. The success of TGMLP U-Net is attributed to the TGMLP structure proposed in this paper, which firstly adopts Global Perceptron to locate the pulmonary infection region roughly and then uses Local Priors to perform further segmentation. This strategy imitates the clinician’s segmentation of infected lung areas from two-dimensional CT images. Besides, we compare Param and FLOPs of TGMLP U-Net on the COVID-19 DataSet. Table2 indicates that the numbers of Param and FLOPs of TGMLP U-Net are minimal, proving that our model can also be applied to smaller devices.

#### Polyp Segmentation DataSet

**Figure 5:**
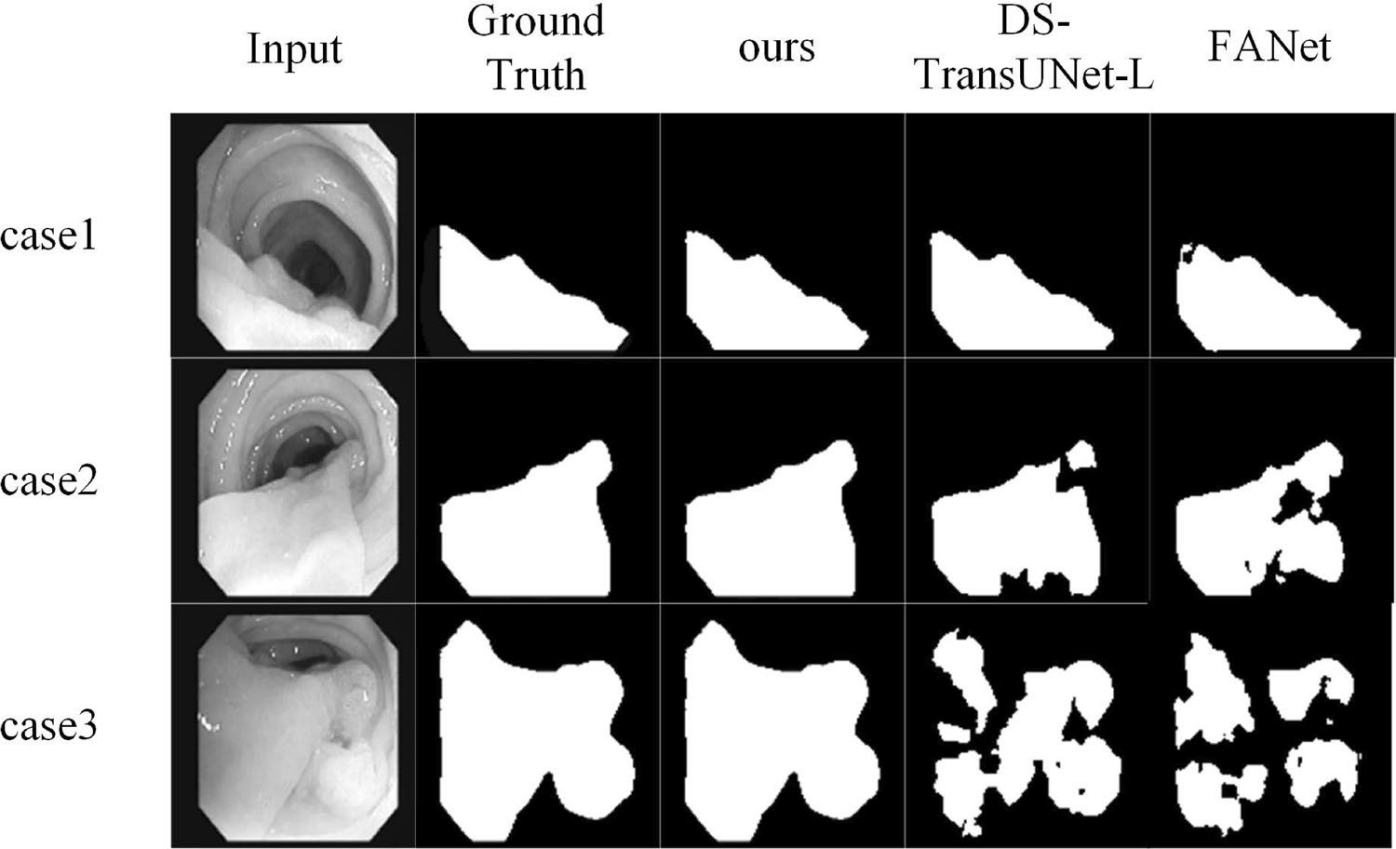
lists the qualitative results of TGMLP U-Net in Polyp Segmentation tasks versus results of other models. Our model with better learning and generalization abilities has better performance.

For the Polyp Segmentation DataSet,evaluation indicators including F1, mIoU, Recall, and Precision are used on the Polyp Segmentation DataSet. Detailed results are shown in Table 3, demonstrating that TGMLP U-Net outperforms the advanced DS-TransUNet-L^29^ and the latest FANet^30^. The values of F1, mIoU, Recall and Precision of TGMLP U-Net increase to 98.21%, 96.57%,97.25% and 95.28%, respectively, which are 3.99%, 7.18%, 2.25%, and 1.59% higher than those of DS-TransUNet-L, respectively. Although DoubleU-Net performs well in Precision, our model produces the best overall performance. As shown in Fig. 5, the visualized results show that our model can accurately predict the position and boundary of colonic polyps and better segment them from normal skin.

#### MoNuSeg DataSet

**Figure 6:**
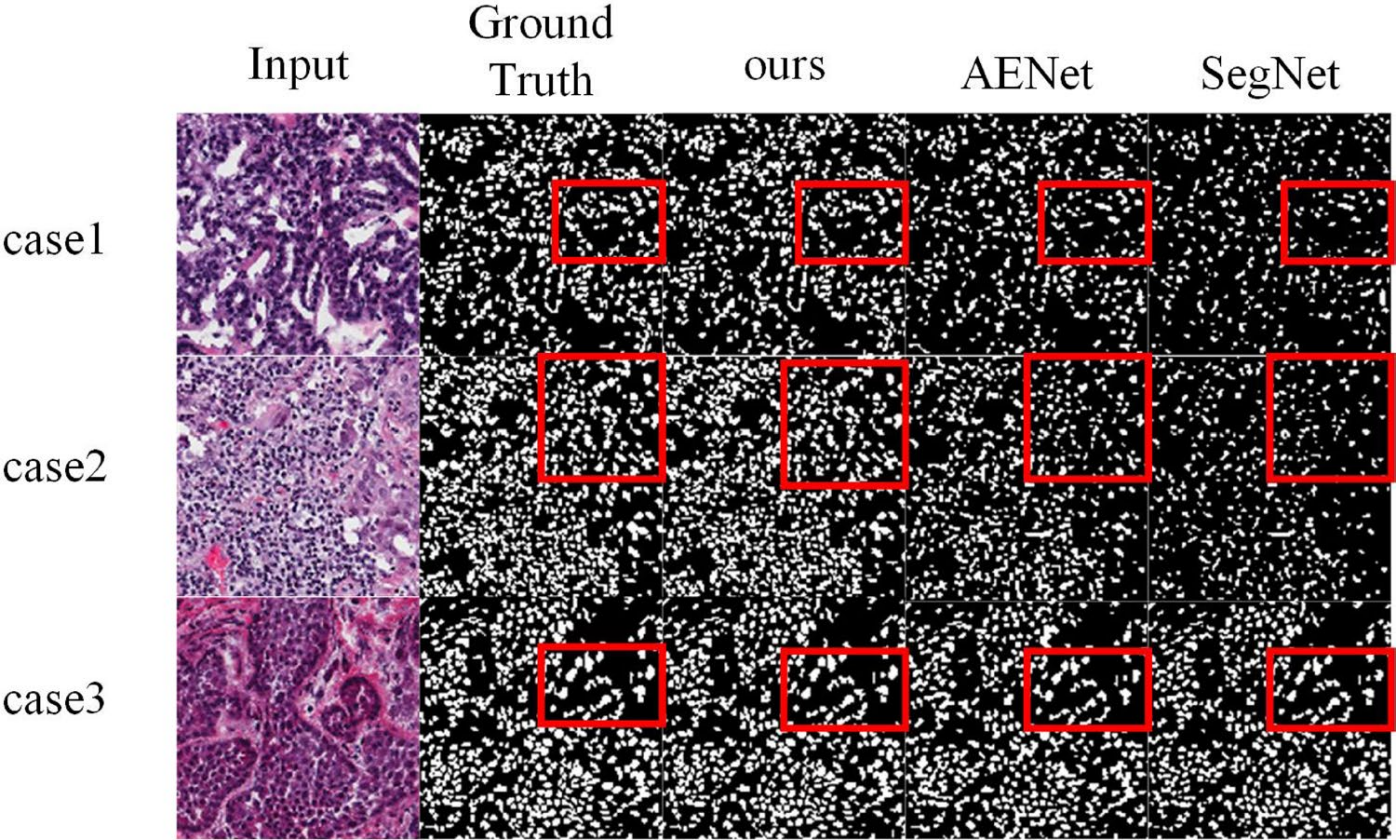
shows the qualitative results of TGMLP U-Net in MoNuSeg task versus results of other models. Our model shows better learning and generalization abilities, resulting in better performance.

We compare our model with other general segmentation models on a small-scale medical DataSet to increase our model’s creditability. MoNuseg is a DataSet with only 30 training samples. The experimental results are listed in Table 4. Compared with AENet^31^, TGMLP U-Net improves mDice by 5.08%, F1 by 1.13%, mIoU by 2.19% and Precision by 1.76%. In addition, we also present the visualized mask image in Fig. 6. It can be noticed that our model can better segment cells from surrounding tissues and has better segmentation performance on the small-scale dataset.

**Table 1:** Shows the experimental results on COVID-19 DataSet. The best values of each indicator are in bold.

Model	mIoU (%)	SEN(%)	SPC(%)	DSC(%)	HD(%)
U-Net^4^	77.56	72.24	97.1	68.37	94.25
FCN-8s^32^	71.85	66.47	93.56	58.11	104.68
FRRN^33^	79.2	78.47	97.5	71.27	86.56
PSPNet^34^	75.61	70.82	96.47	64.55	99.76
Inf-Net^35^	81.62	76.5	**98**.**32**	74.44	86.81
ENet^36^	79.49	81.26	97.53	71.57	96.08
ESPNet^37^	77.45	84.18	96.48	69.3	97.04
CGNet^38^	79.34	81.55	96.34	71.42	90.37
ESPNetv2^39^	78.66	77.84	96.53	70.46	87.77
EDANet^40^	78.74	82.86	96.98	70.67	88.14
LEDNet^28^	77.41	81.59	96.93	68.74	92.49
MiniSeg^27^	82.15	84.95	97.71	75.91	74.42
TGMLP U-Net(ours)	**86**.**45**	**91**.**2**	98.31	**89**.**06**	**50**.**94**

**Table 2:** Shows the comparison of Param, FLOPs on COVID-19 DataSet.

Model	Backbone	Param (M)	FLOPs (G)
PSPNet^34^	ResNet50	64.03	257.79
DeepLabv3^15^	ResNet50	38.71	163.83
DFN^41^	ResNet50	43.53	81.88
EncNet^42^	ResNet50	51.25	217.46
OCNet^43^	ResNet50	51.60	220.69
DANet^44^	ResNet50	64.87	275.72
Inf-Net^35^	ResNet50	30.19	27.30
UNet++^12^	-	8.95	138.37
Attention U-Net^13^	-	8.52	67.14
TGMLP U-Net(ours)	-	**3**.**03**	**1**.**9**

The best values of each indicator are in bold. (“-” indicates that the model does not use the classical deep learning network as the backbone network.)

**Table 3:** Shows the experimental results on Polyp Segmentation DataSet.

Model	F1 (%)	mIoU (%)	Recall (%)	Precision (%)
SFA^45^	70	60.7	–	–
Res-UNet-mod^5^	77.88	45.45	66.83	88.77
UNet++^12^	79.4	72.9	–	–
ResUNet++^46^	79.55	79.62	70.22	87.85
U-Net^4^	82.3	75.5	–	–
PraNet^47^	89.9	84.9	–	–
DoubleU-Net^14^	92.39	86.11	84.57	**95**.**92**
FANet^30^	93.55	89.37	93.39	94.01
DS-TransUNet-L^29^	94.22	89.39	95	93.69
TGMLP U-Net(ours)	**98**.**21**	**96**.**57**	**97**.**25**	95.28

The best values of each indicator are in bold. (“–” indicates that the codes for calculating Recall and Precision are not provided in the source codes offered by the author).

**Table 4:** Evaluation results of MoNuSeg DataSet.

Model	mDice(%)	F1 (%)	mIoU (%)	Precision(%)
Watershed-based^48^	58.3	61.1	57.2	76.3
Fcn^32^	75.2	79.3	72	86.9
U-Net^4^	74.5	76.4	70.5	85.5
FPN^49^	71.6	74.9	68.2	84.4
PSPNet^34^	59.4	64.6	57.9	79.9
SegNet^50^	77.7	81.4	74.3	88.2
AENet^31^	80.4	84.3	73.2	87.8
TGMLP U-Net(ours)	**85.48**	**85.43**	**75.39**	**89.56**

The best results are shown in bold.

### Ablation studies

In this section, to verify the impact of Loss Function on the results, we compare Cross-entropy Loss Function, Dice Loss Function, and Focal Loss Function on the COVID-19 DataSet. The results are shown in Table 5, which illustrates that the results obtained by the Cross-entropy Loss Function are better than those of the Dice Loss Function and Focal Loss Function.

In addition, we implement ablation experiments on COVID-19 DataSet to verify the influence of TGMLP U-Net’s main components, including Local and Global Perceptron (LGP), Triple MLP (TM), Gate-controlled Mechanism (GM) and then visualize the results.

#### The effectiveness of TM

**Figure 7:**
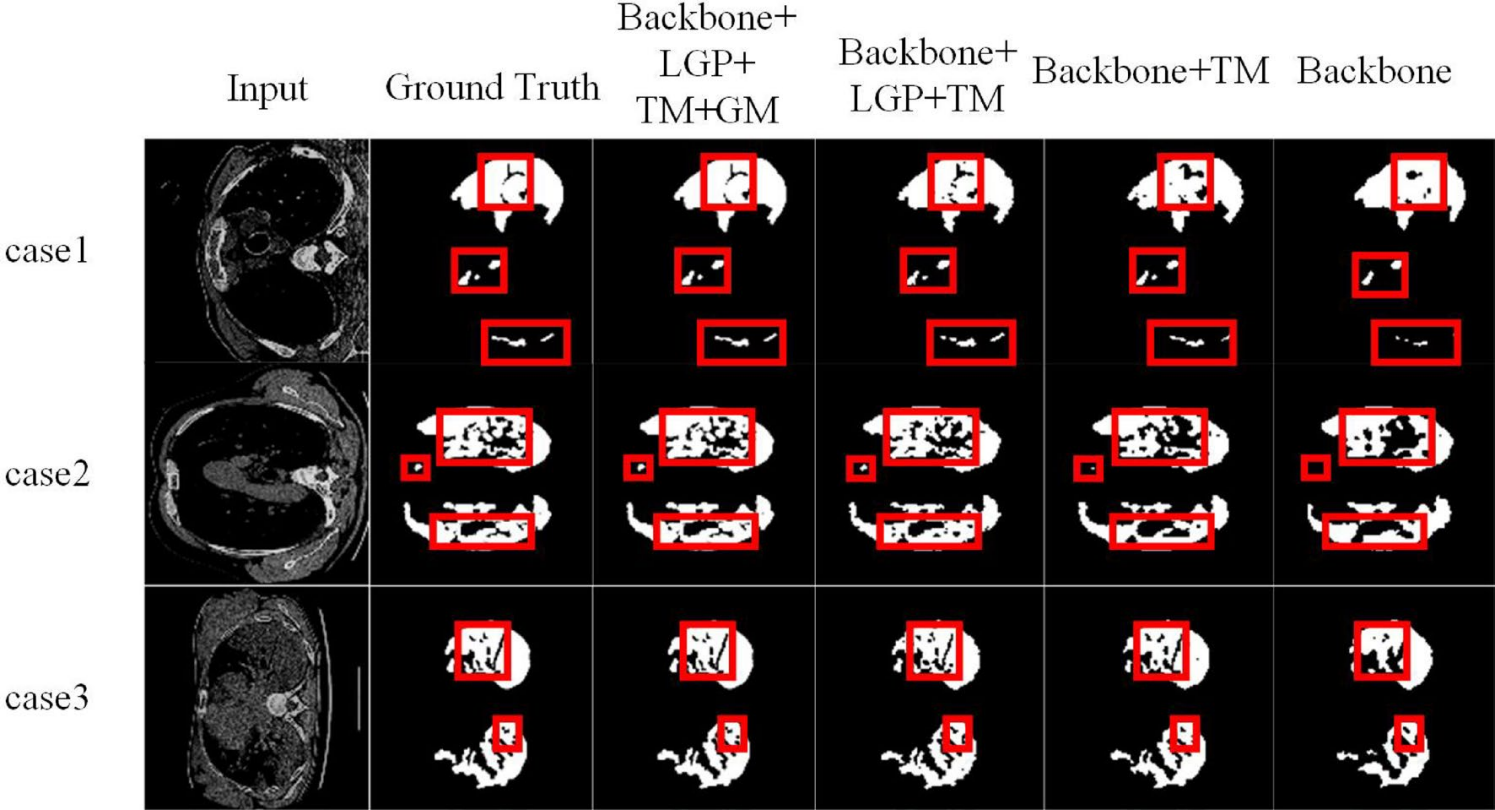
TGMLP U-Net is validated by ablation experiments in COVID-19 tasks, demonstrating the performance of our model’s components.

We explore the importance of TM. With the integration of LGP, all indicators have different increases. From the first and second lines of Table 6, it can be observed that the main indicators including mDice, SEN, SPC and DSC reach 82.3%, 88.4%, 93.1%, 83.2% and 63.9%, respectively. Besides, as can be seen from case l in Fig. 7, the integration of TM enables the model to segment medical images in COVID-19 with the precise position. The main factor of the above performance improvement lies in that TM encodes along the length, wide and high dimensions, making the model generate long-distance dependence between features and improve the position perception ability while preserving the feature structure in the original space dimension of the input feature map.

#### The effectiveness of LGP

We verify the importance of LGP. Compared with line 2, it can be seen from line 3 of Table 6 that the values of mDice, SEN, SPC and DSC increase by 2.2%, 2% and 4.3%, while HD decreases by 8.4%, respectively. Case 2 in Fig. 7 shows that LGP can complementarily extract local and global features. The segmented image contains relatively complete salient objects and preserves the details of the image.

#### The effectiveness of the Gate-controlled Mechanism

We also validate the importance of the Gate-controlled Mechanism. Line 4 of Table 6 shows that the Gate-controlled Mechanism can improve segmentation efficiency. As shown in case 3 of Fig. 7, although LGP and TM can make pixels very close to the segmentation mask, our model learns the dependence between pixel position features better and make LGP and TM find more qualitative features because our method considers the dependence between pixel positions encoded by the Gate-controlled Mechanism.

**Table 5:** Changes of evaluation index values under different Loss Functions.

	Loss functions	mDice (%)	SEN (%)	SPC (%)	DSC (%)	HD (%)
	Cross Entropy Loss	**86.45**	**91.2**	**98.31**	**89.06**	**50.94**
	Focal Loss	85.92	90.82	97.23	88.43	52.35
	Dice Loss	85.39	90.57	96.67	87.89	53.56

The best results are in bold.

**Table 6:** Ablation experiment of TGMLP U-Net model.

Method	mDice (%)	SEN(%)	SPC (%)	DSC (%)	HD (%)
Backbone	79.2	82.3	89.2	81.3	73.2
Backbone+TM	82.3	88.4	93.1	83.2	63.9
Backbone+LGP+TM	84.5	90.4	96.3	87.5	55.5
Backbone+LGP+TM+GM	**86.45**	**91.2**	**98.31**	**89.06**	**50.94**

The best results are shown in bold.

## Conclusion

Triple Gate MultiLayer Perceptron U-Net (TGMLP U-Net), a medical image segmentation model, is proposed in this work, which can segment medical images precisely with less computation overhead. Its performance attributes to three modules: TM, LGP, and a Gate-controlled Mechanism. The TM module encodes coarse-grained and fine-grained feature information from the high, wide, and channel axes, establishing the long-distance dependence between features and outputs sensitive position feature information, significantly improving the model’s performance. In the LGP module, the Global perceptron can be regarded as a sparse full connection with shared parameters, and Local Priors can be regarded as multi-scale Convolution. They can adapt to dynamic features’ changes and complementarily extract local and global features, enabling the model to segment complex medical images. The Gate-controlled Mechanism enables the model to dynamically adapt to DataSets of different sizes, learn the position embedding dependencies between and within Patches, and make the model more sensitive to position information. We use three classical medical image segmentation DataSets to verify the performance of TGMLP U-Net in the experiment, whose results demonstrate its excellent performance in segmenting medical images.

However, our work still has much room for further improvement. For example, whether TGMLP U-Net is suitable for other segmentation tasks or whether it can be reparametrized to accelerate the segmentation speed has not been discussed in detail. In the future, we will continue to study the application of TGMLP U-Net in other image processing tasks, such as denoising, object detection and image super-resolution, to make it applicable to a broader range of computer vision tasks.
